# Integrin β-like 1 protein (ITGBL1) promotes cell migration by preferentially inhibiting integrin-ECM binding at the trailing edge

**DOI:** 10.1007/s13258-021-01204-x

**Published:** 2022-01-23

**Authors:** Dong Gil Jang, Keun Yeong Kwon, Eun Kyung Song, Tae Joo Park

**Affiliations:** 1 Department of Biological Sciences, College of Information-Bio Convergence Engineering, Ulsan National Institute of Science and Technology, Ulsan, 44919 Republic of Korea; 2 School of Medicine, Stanford University, Palo Alto, CA 94305 USA; 3 Center for Genomic Integrity, Institute for Basic Science, Ulsan, 44919 Republic of Korea

**Keywords:** ITGBL1, Integrin, Cell migration, Trailing edge, Focal adhesion

## Abstract

**Background:**

Cell migration is a basic cellular behavior involved in multiple phenomena in the human body such as embryonic development, wound healing, immune reactions, and cancer metastasis. For proper cell migration, integrin and the ECM binding complex must be disassembled for the retraction of trailing edges.

**Objective:**

Integrin must be differentially regulated at leading edges or trailing edges during cell migration. Previously, we showed that ITGBL1 was a secreted protein and inhibits integrin activity. Therefore, we examined the function of ITGBL1 on the retraction of trailing edges during cell migration.

**Methods:**

To examined the function of ITGBL1 on cell migration, we knocked-down or overexpressed ITGBL1 by using ITGBL1 siRNA or ITGBL1 plasmid DNA in human chondrocytes or ATDC5 cells. We then characterized cellular migration and directionality by performing wound healing assays. Also, to analyze leading-edge formation and trailing-edge retraction, we labeled cell membranes with membrane-GFP and performed live imaging of migrating cells and. Finally, we specifically detected active forms of integrin, FAK and Vinculin using specific antibodies upon ITGBL1 depletion or overexpression.

**Result:**

In this study, ITGBL1 preferentially inhibited integrin activity at the trailing edges to promote cell migration. ITGBL1-depleted cells showed increased focal adhesions at the membranous traces of trailing edges to prevent the retraction of trailing edges. In contrast, overexpression of ITGBL1 upregulated directional cell migration by promoting focal adhesion disassembly at the trailing edges.

**Conclusion:**

ITGBL1 facilitates directional cell migration by promoting disassembly of the trailing edge focal adhesion complex.

**Supplementary Information:**

The online version contains supplementary material available at 10.1007/s13258-021-01204-x.

## Introduction

Cell migration is cellular movement toward or away from specific signals and is a basic process of various cell types during embryonic development, wound healing for tissue repair, immune reactions, and even cancer cell metastasis (Friedl and Gilmour 2009; Ridley et al. 2003). Cell migration is initiated in response to specific signals such as secreted proteins, chemicals, or mechanical signals. Cells extend their leading edges using lamellipodia, which provide traction and contractile forces by binding to the extracellular matrix (ECM) to form a focal adhesion complex for cell migration. In contrast, in the rear of cells, trailing edges retract cell membranes by releasing adhesion to the ECM. The cell-ECM interaction is therefore a critical step for both leading edge formation and trailing edge retraction (Ridley et al. 2003; SenGupta et al. 2021).

Integrins, transmembrane proteins consisting of various α- and β-subunit combinations, are well-known key molecules mediating cellular adhesion to the ECM during cell migration. At leading edges, integrins are activated and bind to the ECM and trigger downstream signaling cascades through the cytosolic focal adhesion complex. The focal adhesion complex is comprised of multiple proteins, including focal adhesion kinase (FAK), vinculin, and paxillin, which mediate tractional forces to pull the cell body toward the leading edge. After migration of the main cell body, integrin and the ECM binding complex of the focal adhesion must be disassembled to complete cell migration at the trailing edge of the cell (Hood and Cheresh 2002; Huttenlocher and Horwitz 2011).

It has been previously reported that non-muscle myosin II generates traction forces for trailing edge retraction, and Rho/Rho-kinase-mediated phosphorylation of myosin light chain caused the retraction of the trailing (Ridley et al. 2003). In addition, differential membrane tension allows caveolae to form at the rear of migrating cells, to activate the contractile actin cytoskeleton to promote rapid retraction (Hetmanski et al. 2019). Prior to retraction of the trailing edges, the focal adhesion complex must be disassembled, which is regulated by controlling integrin activity (De Pascalis and Etienne-Manneville 2017; Hood and Cheresh 2002; Huttenlocher and Horwitz 2011; Ridley et al. 2003; SenGupta et al. 2021). However, how integrin and ECM binding is resolved for trailing edge retraction is largely unknown.

Recent studies have reported that integrin β like 1 protein (ITGBL1), which is also called the ten beta integrin EGF-like repeat domain, regulated various forms of cancer cell migration, invasion and metastasis through the WNT/PCP, FAK/SRC, and TGF-β signaling pathways (Berg et al. 1999; Gan et al. 2016; Li et al. 2015; Sun et al. 2016). Furthermore, our previous study showed that ITGBL1 was a secreted protein and that it modulated integrin activity during chondrogenesis (Song et al. 2018). Integrin is a key molecule, which must be differentially regulated at leading edges or trailing edges during cell migration. Therefore, we examined the function of ITGBL1 during cell migration.

In this study, we characterized the behavior of trailing edges upon ITGBL1 knock-down or overexpression during cell migration. ITGBL1-depleted cells showed increased focal adhesions at trailing edges, with retraction of trailing edges being significantly delayed. As a result, ITGBL1 depletion decreased cellular mobility. In the other studies, overexpression of ITGBL1 promoted trailing edge retraction and enhanced cell migration and directionality. Taken together, these findings suggested that ITGBL1 preferentially inhibited the integrin-ECM interaction at the trailing edges and promoted cell migration by facilitating trailing edge retractions.

## Materials and methods

### Cell culture

We purchased human chondrocytes (Cell Application, Inc., San Diego, CA, USA). According to the datasheet provided by the manufacturer, the human chondrocytes were isolated from the human fetal femoral cartilages of a single female donor at 20 weeks gestation. Human chondrocyte cells were cultured in α-minimum essential medium (α-MEM, Sigma-Aldrich, St. Louis, MO, USA) containing 10% fetal bovine serum (FBS; Gibco, Gaitherburg, MD, USA) and 1% antibiotics. Mouse chondrogenic ATDC5 cells were cultured with 5% FBS (Gibco) and 1% antibiotics in DMEM/F12 (1:1) (Gibco). All cells used in this study were transferred to fibronectin-coated coverslips after transfection.

### Transfection

For ITGBL1 siRNA knock-down experiments, human chondrocyte or ATDC5 cells were transfected with siRNA (Genolution, Seoul, Republic of Korea) involving sense, human ITGBL1; 5´-GAGCUGUCUAUGACCGAUAUU-3´, and mouse ITGBL1; 5´-GUGUAAGUGUGAUAAUUCAUU-3´, using jetPRIME® transfection reagent (Polyplus, Berkeley, CA, USA). Exogenous plasmid DNA was transfected using jetPRIME® transfection reagent (Polyplus). All transfections were performed in Opti-MEM® conditions (Gibco). The final concentration of siRNA was 50 nM, and plasmid DNA was transfected with various amounts according to the experimental conditions.

### Immunofluorescence

Cells were fixed with paraformaldehyde fixative solution for 20 min at room temperature. Fixed human chondrocyte cells were incubated in blocking solution (10% FBS, 2% dimethylsuloxide in 1 × TBS with 0.1% Triton X-100) for 30 min at room temperature to block nonspecific binding. Immunostaining was performed with overnight incubations with the following antibodies at 1:300 ~ 1:500 dilutions in blocking solution at 4 °C: anti-integrin β1 (DSHB, Iowa City, IA, USA), anti-active integrin β1 (Millipore, Burlington, MA, USA), anti-total FAK (Abcam, Cambridge, MA, USA), anti-phospho-FAK (pY^397^) (Invitrogen, Carlsbad, CA, USA) and anti-Vinculin (Abcam,). Fluorescence labeling was performed using Alexa Fluor 488- and 555-conjugated secondary antibodies (1:300, Invitrogen).

### Immunoblotting

Human chondrocyte samples were prepared using a lysis buffer (TBS, 10% glycerol, 1% Triton X-100, protease inhibitor, and phosphatase inhibitor (Invitrogen)). After removing cell debris, SDS sample buffer containing dithiothreitol was added. Samples were resolved using SDS-PAGE, and then transferred to PVDF membranes (Merck Millipore). Transferred membranes were incubated in blocking solution (TBS, 0.05% Tween-20 with 5% nonfat powdered milk) for 30 min at room temperature to block nonspecific binding. Immunoblotting was performed using anti-α-actin (Thermo Fisher Scientific, Waltham, MA, USA) and anti-ITGBL1 (AbClon, Seoul, Republic of Korea, custom order) antibodies at 1:1000 ~ 1:3000 dilutions, overnight at 4 °C. The blots were then incubated with horseradish peroxidase-conjugated anti-mouse or anti-rabbit IgG antibodies (both from Thermo Fisher Scientific) at 1:3000 for 1 h at room temperature. Chemiluminescence was performed using SuperSignal™ West Dura Extended Duration Substrate (Thermo Fisher Scientific) and imaged with an iBright imaging system (Thermo Fisher Scientific).

### Wound healing assay

ATDC5 cells were transfected with mouse ITGBL1 siRNA or plasmid DNA. The cells were then transferred to fibronectin-coated 8-well chambers (Lab-Tek™, Thermo Fisher Scientific). Wounds were created by scratching cells with a 10-µL pipette tip. After wound scratching, images of each sample were taken at intervals of approximately 3.5 min during 12 h using a Bio Imaging Navigator microscope (Olympus, Tokyo, Japan).

### Cell migration tracking and data analysis

The migration data were analyzed using the CellTracker program (MATLAB, Natick, MA, USA) (Piccinini et al. 2016). Each cell of each sample was tracked using linear interpolation of a manual tracking method at intervals of three images. Cell tracking data including total displacement, distance from the origin, total movement route, and angle from the origin were quantified. The total movement route figure was created assuming that all cells of each sample started from the same origin, and the angle from the origin figure was created assuming that the scratching area was always located in the right side of the cell layer (cells on the right side of the scratch were assumed to be mirror images). The angle from the origin was plotted by using a MATLAB code that we generated.

### Live imaging

Human chondrocyte cells were transfected with human ITGBL1 siRNA or plasmid DNA with membrane GFP plasmid DNA. The cells were then transferred to fibronectin-coated coverslips on 6-well plates. A total of 78 migration images of each cell from each sample were taken at intervals of approximately 10 min over 7 h using confocal microscopy.

### Microscopy and image analysis

Images were captured using a confocal microscope (LSM780; Zeiss, Jena, Germany) and Bio Imaging Navigator microscopy (FSX100; Olympus). Image analyses were performed using ZEN software (Zeiss) for merging and three-dimensional images. Quantitative analyses of the trailing edge length and area, cell rear boundary length, and percentage area were performed using ImageJ software (National Institutes of Health, Bethesda, MD, USA). P values were calculated using a two-tailed *t* test in Prism 9 software (GraphPad, San Diego, CA, USA).

## Results

### ITGBL1 regulates cellular migration and directionality

**Fig. 1:**
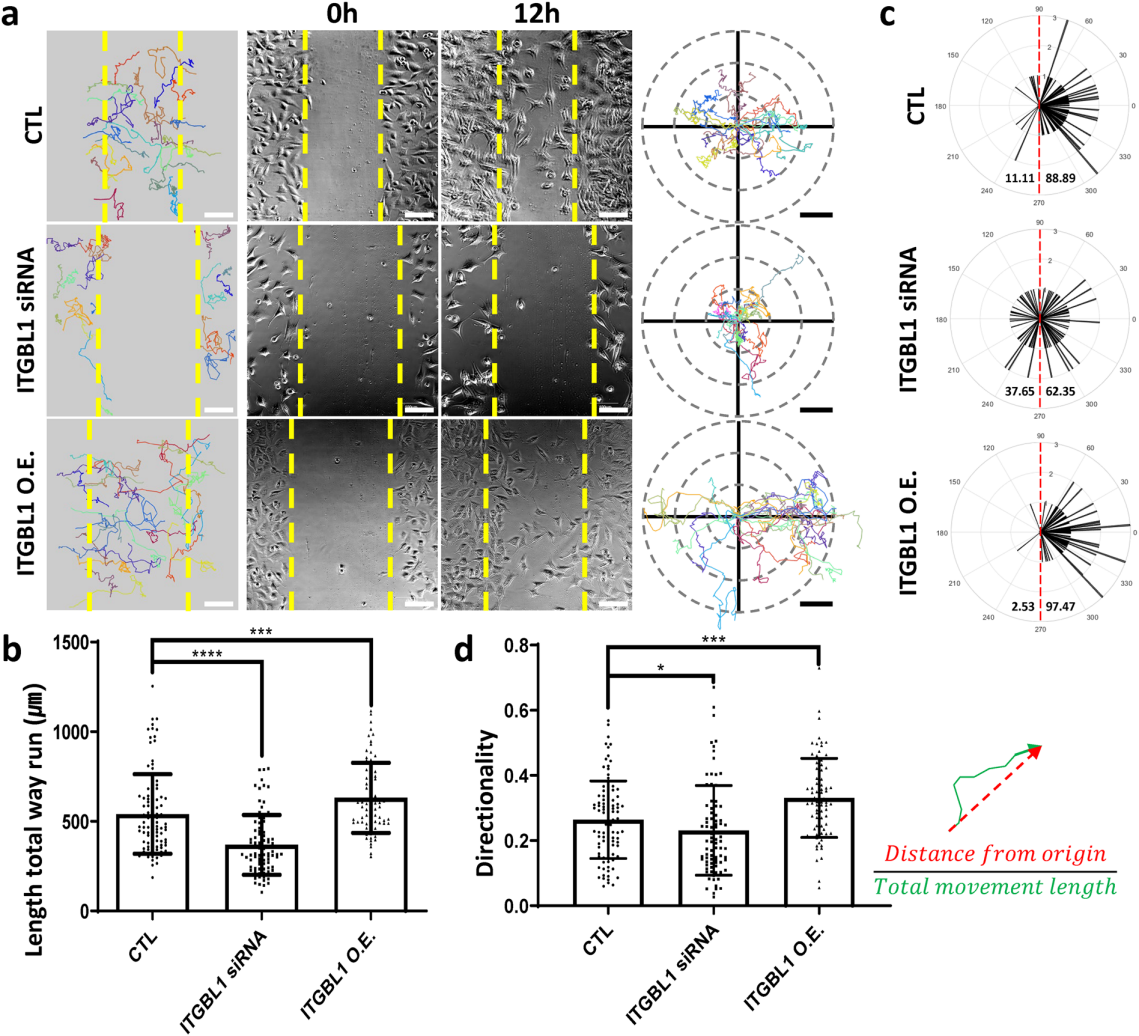
Integrin β-like 1 (ITGBL1) regulates cellular migration and directionality. **a** Wound healing assay analysis of ITGBL1-depleted or -overexpressing cells. Total migration pathways of each cell during 12 h are indicated. Circular graphs show the trajectory of migrating cells from the origin. Scale bars: 100 µm. **b**  Statistical analyses of total length of cell migration trajectory in a. Error bars represent the mean with the SD. P values were determined using the two-tailed t test (^****^P < 0.0001; ^***^P = 0.0005). **c** The angles between the origin and the final position of each cell were quantified and plotted. Cells on the right side of the scratch were assumed to be mirror images to match the angle between both sides of the wound. **d** Statistical analyses of the directionality of cell migration. The directionality was calculated using the ratio of distance from origin to the total length of the trajectory. Error bars represent the mean with SD. P values were determined using the two-tailed *t* test (^*^P = 0.0214; ^***^P = 0.0003)

Previously, ITGBL1 was reported to promote metastasis in various types of cancers by controlling either Wnt-PCP signaling or the FAK/SRC or TGF-β signaling pathways (Berg et al. 1999; Gan et al. 2016; Li et al. 2015; Sun et al. 2016). However, ITGBL1 is known to be a secreted protein, and we have experimentally shown that ITGBL1 was a secreted inhibitor of integrin (Song et al. 2018). Integrin is critical for cell migration and mediates cell-ECM interactions. We therefore determined how ITGBL1 promoted cell migration by inhibiting integrin activation. To achieve this objective, we knocked-down or overexpressed ITGBL1 by using ITGBL1 siRNA or ITGBL1 plasmid DNA in human chondrocytes or ATDC5 cells, which highly expressed ITGBL1. ITGBL1 expression was effectively decreased or increased in the siRNA or ITGBL1 cDNA transfected cells respectively (Fig. S1a). We then characterized cellular migration and directionality by performing wound healing assays. As previously reported, ITGBL1-depleted cells showed decreased cellular migration, when compared with control cells (Fig. 1a, b). In contrast, ITGBL1-overexpressed cells showed increased cell migration compared to control cells (Fig. 1a, b). Cellular directionality was also affected by ITGBL1 expression. ITGBL1-depleted cells showed reduced directionality, and ITGBL1 overexpression resulted in higher directionality, when compared with control cells (Fig. 1c, d). Together, these results indicate that ITGBL1 promotes migration and is also necessary for directional cellular movement.

### ITGBL1 regulates trailing edge retraction at the rear of migrating cells

**Fig. 2:**
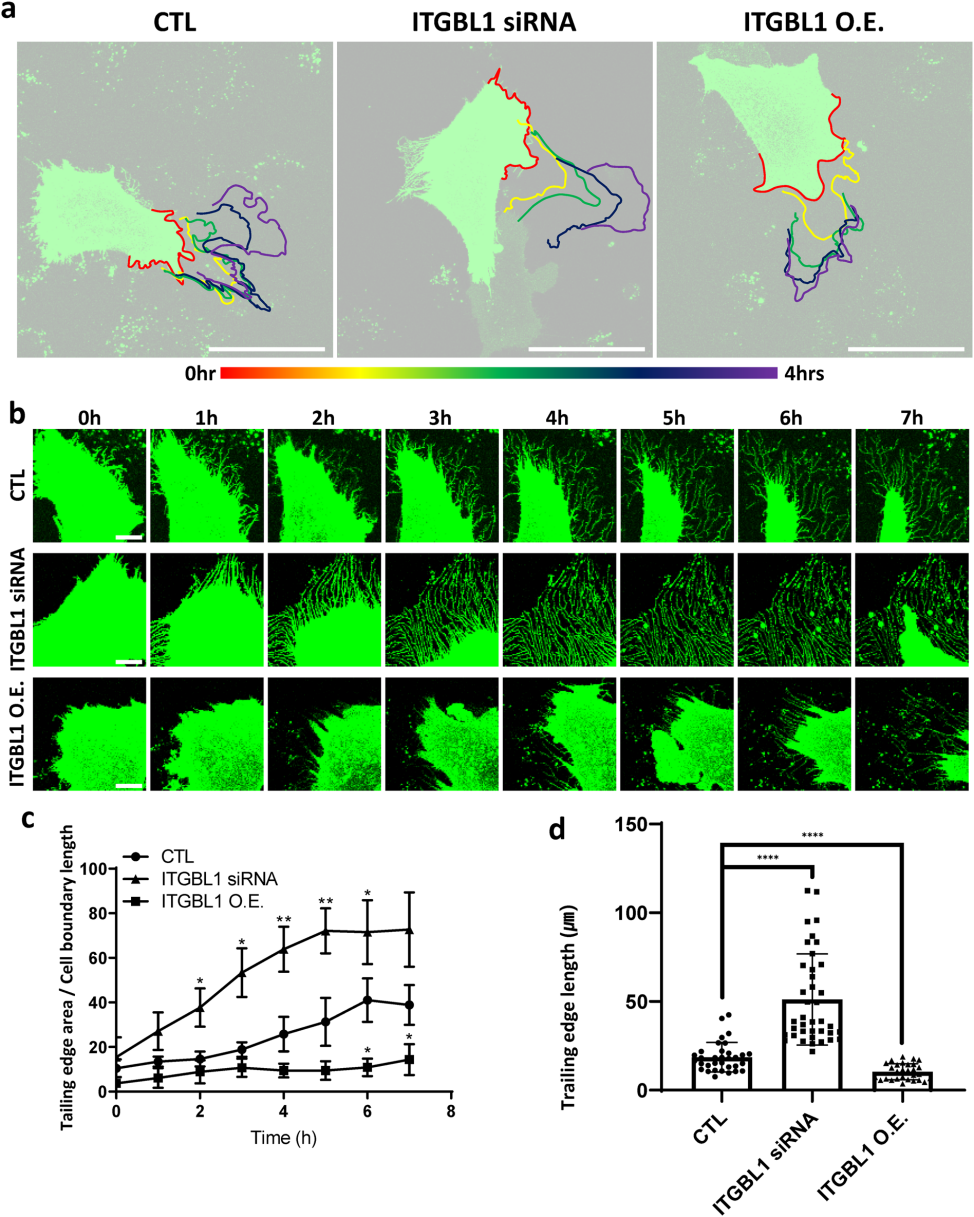
Integrin β-like 1 (ITGBL1) regulates trailing edge retraction at the rear of migrating cells. **a** Leading edge behaviors over 4 h in migrating cells were indicated by different colors. Scale bars: 50 µm. **b** Trailing edge time-lapse live images of ITGBL1-depleted or overexpressed cells over 7 h. Scale bars: 10 µm. **c** Statistical analysis of the ratio of trailing edge areas to the cell boundary length, from which the trailing edge originates. Error bars represent the mean with SD. *P* values were determined using the two-tailed *t* test (^*^P < 0.05; ^**^P < 0.01). **d** Statistical analyses of the trailing edge lengths. Error bars represent the mean with the SD. *P* values were determined using the two-tailed *t*-test (^****^P < 0.0001)

Next, we characterized a possible molecular mechanism involving how ITGBL1 may have promoted both migration and directionality during cell migration. We labeled cell membranes with membrane-GFP and performed live imaging of migrating cells, followed by analyses of leading-edge formation and trailing-edge retraction (Video S1–3). To our surprise, ITGBL1 depletion did not significantly affect the dynamics of lamellipodia at the leading edge (Fig. 2a). Instead, ITGBL1 depletion dramatically increased the traces of trailing edges (Fig. 2b). To better analyze the striking phenotypes of ITGBL1 depletion at trailing edges, we measured the total areas and the lengths of membranous extensions of trailing edges. The results showed sharp increases of membranous protrusions at the trailing edges in the ITGBL1-depleted cells, while overexpression of ITGBL1 significantly decreased the membranous extensions of trailing edges (Fig. 2b–d).

Furthermore, in ITGBL1-depleted cells, the membranous tracts of trailing edges were retained, and the migrating cells failed to retract membranous extensions of trailing edges, even after complete migration of cell bodies. As a result, total cell bodies were retracted backward due to the retention of trailing edge membranes (Sup. Movie 1). Taken together, these results show that ITGBL1 promoted cell migration by preferentially affecting trailing edge retraction.

### ITGBL1 regulates trailing edge retraction by inhibiting integrin activation

**Fig.3:**
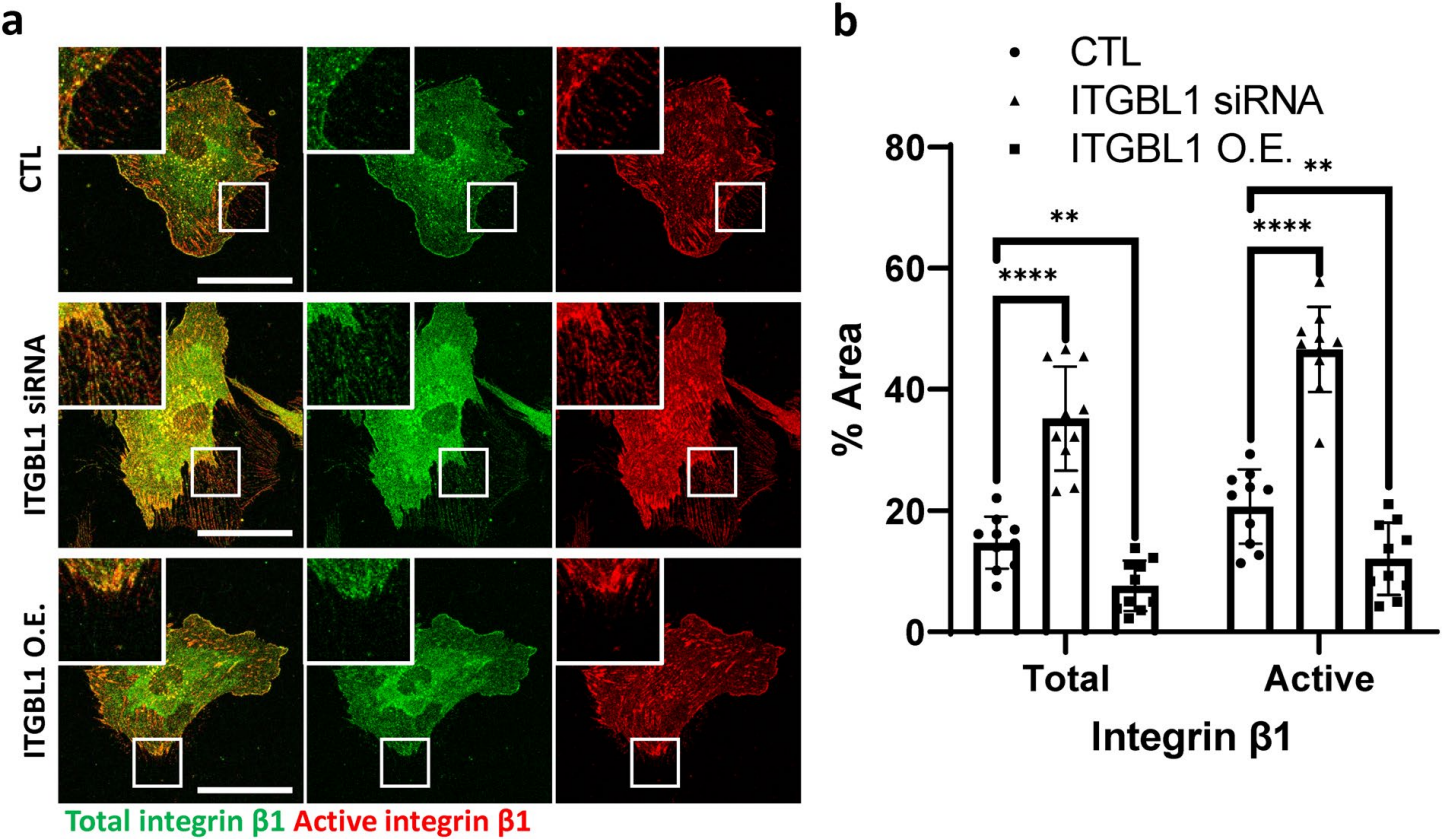
Integrin β-like 1 (ITGBL1) regulates trailing edge retraction by inhibiting integrin activation. **a** Confocal images of total or active integrin β1 in ITGBL1-depleted or overexpressed cells. Enlarged inlet images are indicated by white rectangles. Scale bars: 50 µm. **b** Statistical analysis of total or active integrin. The area of fluorescent signals was normalized using the total areas of trailing edges. Error bars represent the mean with the SD. *P* values were determined using the two-tailed *t* test (^****^P < 0.0001; ^**^P < 0.01)

We reasoned that the integrin-ECM interactions must be downregulated during trailing edge retraction, and that ITGBL1 may inhibit integrin activation at the trailing edges. We therefore determined whether integrin activation was regulated by the expression level of ITGBL1 during trailing edge retraction. To achieve this objective, we specifically detected active forms of integrin using the active integrin specific antibody upon ITGBL1 depletion or overexpression. As expected, we observed that total integrin β1 levels were dramatically increased at the trailing edges of ITGBL1-depleted cell (Fig. 3a, b). Furthermore, active forms of specific immunofluorescent integrin β1 were also significantly increased in the trailing edges of ITGBL1-depleted cells (Fig. 3a, b). In contrast, active forms of integrin β1 signals were significantly decreased in ITGBL1-overexpressed trailing edges (Fig. 3a, b). Overall, these results strongly support the idea that ITGBL1 promotes retraction of trailing edge membranes by preferentially inhibiting integrin activation at the trailing edges.

### ITGBL1 mediated-integrin inhibition is necessary for focal adhesion disassembly for trailing edge retraction

**Fig. 4:**
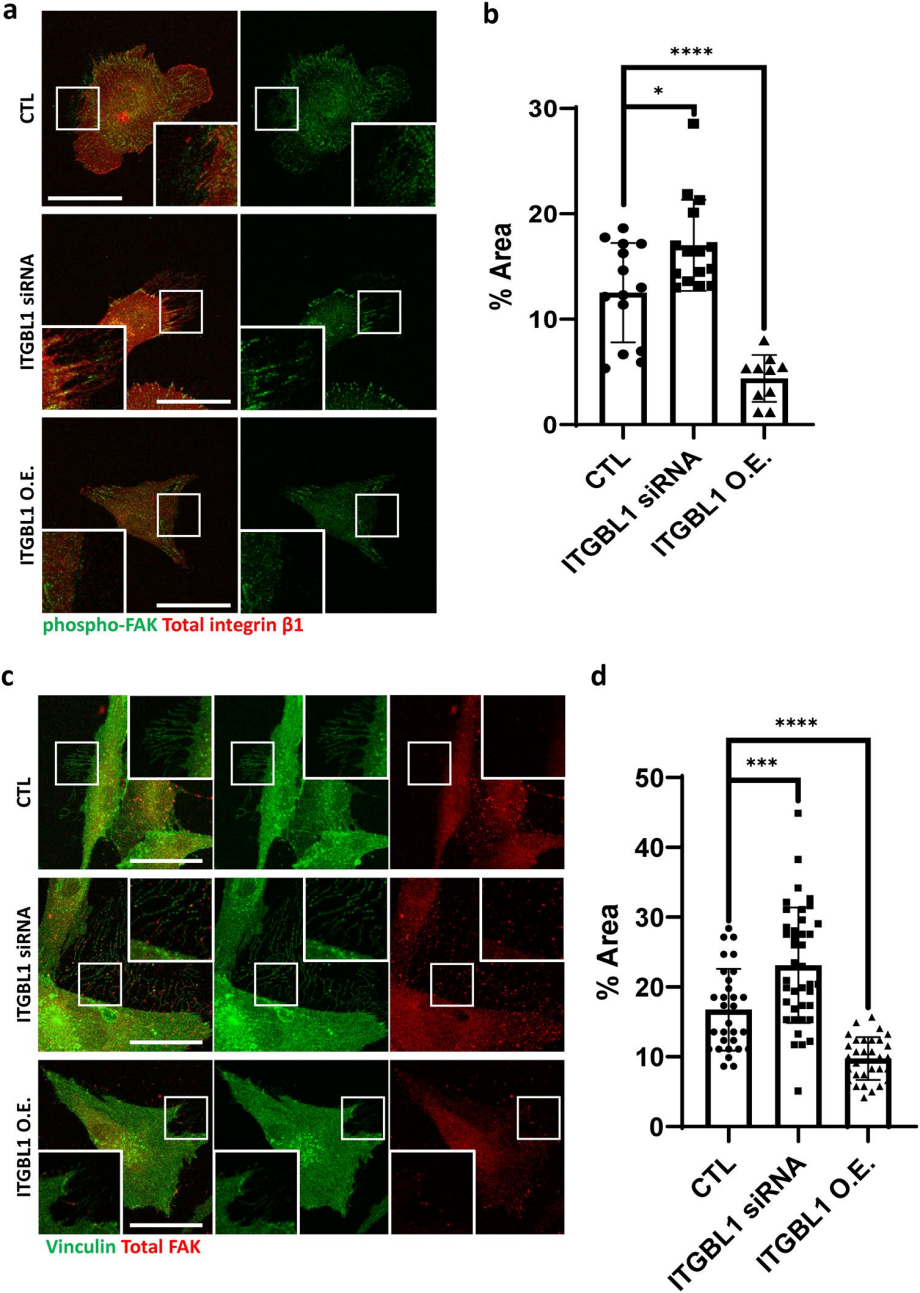
Integrin β-like 1 (ITGBL1)-mediated-integrin inhibition is necessary for focal adhesion disassembly for trailing edge retraction. **a** Confocal images of phospho-FAK in ITGBL1-depleted or overexpressed cells. Enlarged inlet images are indicated by white rectangles. Scale bars: 50 µm **b** Statistical analysis of phospho-FAK. The areas of fluorescent signals were normalized using the total areas of trailing edges. Error bars are the mean with the SD. *P* values were determined using the two-tailed *t* test (^*^P = 0.0377; ^****^P < 0.0001). **c** Confocal images of total-FAK and vinculin in ITGBL1-depleted or overexpressed cells. Enlarged inlet images are indicated by white rectangles. Scale bars: 50 µm. **d** Statistical analysis of vinculin. The areas of fluorescent signals were normalized using the total areas of trailing edges. Error bars represent the mean with the SD. *P* values were determined using the two-tailed *t* test (^***^P = 0.0004; ^****^P < 0.0001)

Activated integrins, which are bound with the ECM, assemble focal adhesion complexes, which are connected to microfilaments and trigger the downstream signaling cascade. FAK are recruited to the cytoplasmic domain of ECM-bound integrins and activated by phosphorylation. FAK is one of the master regulators for numerous cellular functions, and broadly regulates multiple downstream pathways (Guan 2010). We therefore analyzed FAK recruitment to trailing edge membranes by using immunofluorescent imaging. As expected, ITGBL1 depletion significantly increased the localization of phosphorylated FAK at membranous traces at the trailing edges (Fig. 4a, b). In contrast, ITGBL1 overexpressed cells showed significantly decreased phospho-FAK signals (Fig. 4a, b). Vinculin is also a well-known marker of focal adhesion complex, which mediates binding between focal adhesion complex and microfilaments (Katoh 2020). Therefore, we also measured vinculin localization by using immunofluorescent imaging, and consistently observed that ITGBL1 depletion increased vinculin signals at the trailing edges and vinculin signals were significantly decreased in ITGBL1-overexpressed trailing edges (Fig. 4c, d). Together, these results suggest that ITGBL1 mediated-integrin inhibition was necessary for focal adhesion disassembly for trailing edge retraction.

## Discussion

Cell migration is among the most basic processes of various cellular functions such as wound healing for tissue repair, immune reactions, and cancer cell metastasis. During cell migration, cells extend lamellipodia for traction and contractile forces at the leading edges, while the trailing edges must be retracted for directional cell migration. Cell-ECM interactions must therefore be differentially regulated at the leading and trailing edges.

Integrins are key molecules mediating cellular adhesion to the ECM during cell migration. At the leading edges, integrins bind to the ECM and mediate traction forces for cell migration. In contrast, integrin-ECM binding and focal adhesion need to be disassembled for trailing edge retraction, which is critical for directional cell migration.

The molecular mechanisms controlling leading edge dynamics are relatively well understood, while the retraction of trailing edges has been less characterized. A recent study suggested that the Rho/Rho-kinase/non-muscle myosin II cascade and differential membrane tension generated traction forces necessary for retraction of the trailing edges (Ridley et al. 2003). However, how the integrin and ECM binding is resolved during trailing edge retraction is largely unknown.

ITGBL1 is known to promote cancer metastasis and invasion by the WNT/PCP, FAK/SRC, and TGF-β signaling pathways, yet our previous study suggested that ITGBL1 was a secreted protein and that it inhibited integrin activation (Berg et al. 1999; De Pascalis and Etienne-Manneville 2017; Gan et al. 2016; Li et al. 2015; Sun et al. 2016). In the present study, we identified the function of ITGBL1 during trailing edge retraction, which showing that ITGBL1 preferentially inhibits trailing edge integrin activation and promoted trailing edge retraction. Unfortunately, we were not able to discover a molecular mechanism by which ITGBL1 preferentially regulates trailing edge integrins. One of the possible mechanisms could be the accessibility of ITGBL1 to the focal adhesion complex. It has been shown that active Integrin-ECM complexes form clusters by integrin homo-oligomerization (Li et al. 2001). We suspect that the leading edge integrins are not easily accessible by ITGBL1 due to the integrin clustering, thereby trailing edge integrins may preferentially inactivated by ITGBL1. Nonetheless, we believe preferential inhibition of trailing edge integrins by ITGBL1 is a novel mechanism explaining how integrin inhibition may promote cell migration during cancer cell invasion and metastasis, where ITGBL1 has been shown to be a key regulator.

## Supplementary Information

Below is the link to the electronic supplementary material.

Supplementary file1 (DOCX 48 KB)

Supplementary file2 (AVI 11427 KB)

Supplementary file3 (AVI 11427 KB)

Supplementary file4 (AVI 11427 KB)

## Data Availability

The data and materials in the current study are available on request.

## Abbreviations

ITGBL1: Integrin β-like 1
ECM: Extracellular matrix
FAK: Focal adhesion kinase

## References

[CR1] Berg RW, Leung E, Gough S, Morris C, Yao WP, Wang SX, Ni J, Krissansen GW (1999). Cloning and characterization of a novel beta integrin-related cDNA coding for the protein TIED ("ten beta integrin EGF-like repeat domains") that maps to chromosome band 13q33: a divergent stand-alone integrin stalk structure. Genomics.

[CR2] De Pascalis C, Etienne-Manneville S (2017). Single and collective cell migration: the mechanics of adhesions. Mol Biol Cell.

[CR3] Friedl P, Gilmour D (2009). Collective cell migration in morphogenesis, regeneration and cancer. Nat Rev Mol Cell Biol.

[CR4] Gan X, Liu Z, Tong B, Zhou J (2016). Epigenetic downregulated ITGBL1 promotes non-small cell lung cancer cell invasion through Wnt/PCP signaling. Tumour Biol.

[CR5] Guan JL (2010). Integrin signaling through FAK in the regulation of mammary stem cells and breast cancer. IUBMB Life.

[CR6] Hetmanski JHR, de Belly H, Busnelli I, Waring T, Nair RV, Sokleva V, Dobre O, Cameron A, Gauthier N, Lamaze C (2019). Membrane tension orchestrates rear retraction in matrix-directed cell migration. Dev Cell.

[CR7] Hood JD, Cheresh DA (2002). Role of integrins in cell invasion and migration. Nat Rev Cancer.

[CR8] Huttenlocher A, Horwitz AR (2011). Integrins in cell migration. Cold Spring Harb Perspect Biol.

[CR9] Katoh K (2020) FAK-dependent cell motility and cell elongation. Cells 9 10.3390/cells9010192 PMC7017285 31940873

[CR10] Li R, Babu CR, Lear JD, Wand AJ, Bennett JS, DeGrado WF (2001). Oligomerization of the integrin alphaIIbbeta3: roles of the transmembrane and cytoplasmic domains. Proc Natl Acad Sci USA.

[CR11] Li XQ, Du X, Li DM, Kong PZ, Sun Y, Liu PF, Wang QS, Feng YM (2015). ITGBL1 Is a Runx2 transcriptional target and promotes breast cancer bone metastasis by activating the TGFbeta signaling pathway. Cancer Res.

[CR12] Piccinini F, Kiss A, Horvath P (2016). Cell Tracker (not only) for dummies. Bioinformatics.

[CR13] Ridley AJ, Schwartz MA, Burridge K, Firtel RA, Ginsberg MH, Borisy G, Parsons JT, Horwitz AR (2003). Cell migration: integrating signals from front to back. Science.

[CR14] SenGupta S, Parent CA, Bear JE (2021). The principles of directed cell migration. Nat Rev Mol Cell Biol.

[CR15] Song EK, Jeon J, Jang DG, Kim HE, Sim HJ, Kwon KY, Medina-Ruiz S, Jang HJ, Lee AR, Rho JG et al (2018) ITGBL1 modulates integrin activity to promote cartilage formation and protect against arthritis. Sci Transl Med 10 10.1126/scitranslmed.aam7486 30305454

[CR16] Sun L, Wang D, Li X, Zhang L, Zhang H, Zhang Y (2016). Extracellular matrix protein ITGBL1 promotes ovarian cancer cell migration and adhesion through Wnt/PCP signaling and FAK/SRC pathway. Biomed Pharmacother.

