# Supplementary material for: Integrin β-like 1 protein (ITGBL1) promotes cell migration by preferentially inhibiting integrin-ECM binding at the trailing edge
Source: Genes Genomics. 2022 Jan 23;44(4):405–13. doi: 10.1007/s13258-021-01204-x (PMC8921176; doi:10.1007/s13258-021-01204-x)
Supplement: Supplementary file 1 — Supplementary file1 (DOCX 48 KB) [file 13258_2021_1204_MOESM1_ESM.docx]

**Supplementary information**

**Integrin β-like 1 protein (ITGBL1) promotes cell migration by preferentially inhibiting integrin-ECM binding at the trailing edge**

Dong Gil Jang^a†^, Keun Yeong Kwon^a†^, Eun Kyung Song^b*^ and Tae Joo Park^a,c*^

^a^Department of Biological Sciences, College of Information-Bio Convergence Engineering, Ulsan National Institute of Science and Technology, Ulsan 44919, Republic of Korea

^b^School of Medicine, Stanford University, Palo Alto, CA, USA 94305

^c^Center for Genomic Integrity, Institute for Basic Science, Ulsan 44919, Republic of Korea

^†^The authors contributed equally to this study.

^*^Corresponding authors: parktj@unist.ac.kr, eksong@stanford.edu

**Supplementary Figure 1**


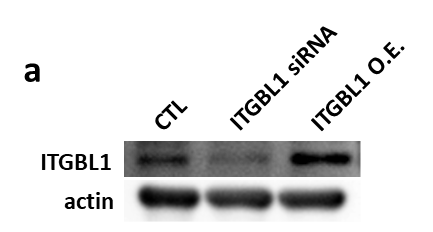


**a.** ITGBL1 expression was knocked-down or overexpressed by using ITGBL1 siRNA or ITGBL1 plasmid DNA in human chondrocytes and the expression level was analyzed by performing western blot analysis.

**Supplementary Video 1**

The cell membrane of control human chondrocytes was labeled with membrane-GFP for live imaging. The live imaging of the cells was performed for 7 hours with 10 min interval.

**Supplementary Video 2**

The cell membrane of ITGBL1-siRNA transfected human chondrocytes was labeled with membrane-GFP for live imaging. The live imaging of the cells was performed for 7 hours with 10 min interval.

**Supplementary Video 3**

The cell membrane of ITGBL1 cDNA transfected human chondrocytes was labeled with membrane-GFP for live imaging. The live imaging of the cells was performed for 7 hours with 10 min interval.
